# IgE Levels to *Ascaris* and House Dust Mite Allergens Are Associated With Increased Histone Acetylation at Key Type-2 Immune Genes

**DOI:** 10.3389/fimmu.2020.00756

**Published:** 2020-04-28

**Authors:** Josefina Zakzuk, Nathalie Acevedo, Hani Harb, Lisa Eick, Harald Renz, Daniel P. Potaczek, Luis Caraballo

**Affiliations:** ^1^Institute for Immunological Research, University of Cartagena, Cartagena, Colombia; ^2^Institute of Laboratory Medicine, Member of the German Center for Lung Research (DZL), Universities of Giessen and Marburg Lung Center (UGMLC), Philipps-University Marburg, Marburg, Germany; ^3^John Paul II Hospital, Krakow, Poland

**Keywords:** histone acetylation, IgE levels, nematode infection, H3Ac, H4Ac, house dust mites, epigenetics

## Abstract

**Background:**

Epigenetic changes in response to allergen exposure are still not well understood. The aim of this study was to evaluate histone acetylation levels in peripheral blood leukocytes from humans naturally infected by intestinal parasites and perennially exposed to house dust mites (HDM).

**Methods:**

Peripheral blood mononuclear cells (PBMCs) were isolated by gradient centrifugation from 20 infected and 21 non-infected individuals living in a rural/village in Colombia. Histone 3 acetylation (H3Ac) and histone 4 acetylation (H4Ac) levels were measured in six immune genes previously associated with helminth immunity by chromatin immunoprecipitation (ChIP)-quantitative PCR. Then we analyzed the association between histone acetylation levels with total parasite egg burden and IgE levels.

**Results:**

We found an inverse correlation between H4Ac levels in the *IL13* gene and egg worm burden that remained significant after adjustment by age [−0.20 (−0.32 to −0.09), *p* < 0.0001]. Moreover, we found significant associations between H4Ac levels in *IL4* [0.32 (0.05–0.60), *p* = 0.02] and *CHI3L1* [0.29 (0.08–0.51), *p* = 0.008] with the IgE levels to *Ascaris lumbricoides*. In addition, the levels of specific IgE antibodies to HDM were associated with H4Ac levels in the gene *TNFSF13B* encoding the B cell activating factor (BAFF) [0.51 (0.26–0.76), *p* < 0.001]. All values are presented as beta (95% CI).

**Conclusion:**

Histone acetylation levels at key type-2 immune genes in humans were modified by nematode infection and HDM allergens and are associated with the intensity of the IgE response.

## Introduction

Epigenetic modifications and more specifically DNA methylation, have been associated with increased total IgE levels (1) and increased IgE sensitization to house dust mites (HDM) (2). In addition, allergen exposure induces epigenetic changes in immune cells affecting the inception and maintenance of type-2 skewed immune phenotypes (3). The IgE response to HDM allergens is very prominent in humans living in tropical environments, and even though perennial exposure may explain this observation, the co-exposure with intestinal helminth infection provides a unique opportunity to dissect key molecular events implicated in type 2 immunity (4). Indeed, a study in a mice model revealed that chronic helminth infection also reprograms T cell differentiation via histone acetylation changes (5), by the addition of acetyl groups to lysine residues (K) at the N-terminal tail of histones. Acetylation neutralizes the positive charge of lysine reducing histone affinity for DNA and (this way) opens chromatin. Also, by providing a tag in histone tails for transcription factors and regulatory proteins, histone acetylation affects the accessibility of promoters to the transcriptional machinery (6, 7). Several studies support that H3 acetylation at K9 and K14 (H3Ac) are a hallmark of gene activation and exhibit remarkable correlation with active promoters and active enhancers. Also, H4 acetylation has been associated with transcriptional activation and maintenance of euchromatin. Therefore, increased acetylation of lysine residues at H3 and H4 is informative on active transcription of the marked gene (6, 7).

Previous studies revealed that when naïve T cells differentiate into Th2 cells, the Th2 locus (chromosome 5q31) undergoes extensive epigenetic modifications that lead to a poised chromatin configuration (8), making chromatin accessible and promoting IL-4, IL-5, and IL-13 expression (9, 10). Regulatory regions, such as the *IL4* and *IL13* promoters, the Th2 locus control region (LCR), and enhancers are the primary targets of these modifications (11–13). The isotype class switching and specific IgE production resulting from these changes may be used as a proxy of Th2 locus activation. In the context of helminth infection, the magnitude of IgE production to parasite components depends on the individual predisposition toward type 2 immunity (14–17). Egg burden is also a marker of individual ability to resist parasite infection (18, 19). A quantitative trait locus (QTL) for *Ascaris* egg counts has been described in chromosome 13q33 in a region encoding for ligase IV (*LIG4*), abhydrolase domain containing 13 (*ABDH13*) and the B cell activating factor BAFF (*TNFSF13B*) (20). Genetic variants in this region are also associated with increased IgE against *Ascaris* and the HDM *Dermatophagoides pteronyssinus* (21), although the underlying mechanisms remain unclear. Since parasite immunity and allergic responses share several biological pathways, we hypothesized that the relative effects of these genes depend on environmental factors that could induce epigenetic modifications.

To date, no study has analyzed if exposure to *Ascaris lumbricoides* and HDM allergens can influence histone acetylation at these loci. In this study, we aimed to evaluate H3 and H4 acetylation levels in mononuclear leukocytes from humans living in a rural community exposed to *A. lumbricoides* and HDM, and to investigate the relationship of H3 and H4 acetylation with the specificity and intensity of the IgE response.

## Materials and Methods

### Study Population

For this study we selected 41 subjects from a cohort of 739 well-characterized subjects living in Santa Catalina (Colombia) and previously described by Zakzuk et al. (20). This is a small tropical farming/fishing town in northern Colombia (10° 36′ 0″ N, 75° 18′ 0″ W) with a territorial extension of 153 km^2^ and a population of approximately 12,500 inhabitants. Half of the people have at least one unsatisfied basic need, only 4.5% of the population has a sewage system and 56% has tap water. This study included 20 non-infected subjects and 21 infected with *A. lumbricoides* (Table 1). Criteria for non-infected subjects included having two negative results in stool examinations conducted in 2014 (22), and when resampled in two consecutive stool tests collected for this study during May–June 2016. Criteria for infected subjects include active parasite infection as detected by fresh fecal smear in at least one stool test collected for this study in 2016. Parasite burden was quantified as eggs per gram (e.p.g) of feces by the Kato Katz method using a commercial kit (Copro Kit, C&M Medical, Campinas, Brazil). Blood samples were taken on the same day or within 2 days after the stool test. Albendazole treatment was prescribed after blood sampling in all infected subjects. This study was approved by the Ethics Committee of the University of Cartagena (nr. 1705-2012) and was conducted following the guidelines of the Declaration of Helsinki. All the participants gave their written informed consent prior to their inclusion in the study.

**TABLE 1:** Descriptive features of the study sample according to *Ascaris* infection status.

	Infected (*n* = 21)	Non-infected (*n* = 20)	*p*-value
Age (mean ± SD)	23.8 ± 18.7	33.7 ± 19.2	0.053
Female [*n* (%)]	12 (57.1)	13 (65)	0.7
*Trichuris* epg. [median (IQR)]	2.244 (655−8150)	0 (0−0)	n/a
*Ascaris* epg. [median (IQR)]	8.020 (2015−8940)	0 (0−0)	n/a
Total egg burden [median (IQR)]	9030 (3689−16820)	0 (0−0)	n/a
IgE levels, kU/L [GM ± SD]			
*Ascaris* spp.	1.84 ± 5.9	0.31 ± 1.9	0.004
*D. pteronyssinus*	0.37 ± 13.7	0.24 ± 4.3	0.1
*B. tropicalis*	0.72 ± 26.3	0.29 ± 13.6	0.3
IgE ≥ 0.35 kU/l [*n* (%)]			
*Ascaris* spp.	17 (81)	10 (50)^†^	0.037
*D. pteronyssinus*	8 (38.1)	7 (35)	0.8
*B. tropicalis*	11 (52.4)	7 (35)	0.2
Total IgE, kU/L [GM ± SD]	736 ± 1207	176 ± 568	0.003

*epg: eggs per gram of feces; GM: geometric mean; SD: standard deviation. ^†^Due to immune memory, non-infected subjects can be found positive for IgE antibodies to Ascaris albeit not having active infection by the time of blood sampling.*

### Isolation of Peripheral Blood Mononuclear Cells (PBMCs)

Blood samples were collected in EDTA tubes by standard phlebotomy and 3 mL of blood were mixed with 3 mL of RPMI-1640 based-medium (RPMI-1640 supplemented with 10% heat-inactivated Fetal Calf Serum (FCS), 1% antibiotic/antimycotic solution and 1% L-Glutamine) and then, layered over 3 mL of Histopaque (Sigma, city, United States). The sample was centrifuged at 800 × *g* for 20 min without a break. The mononuclear cell layer was aspirated, transferred to a new tube, and resuspended in 10 mL of RPMI-1640 based-medium. Cells were washed at 800 *g* for 10 min and the cell pellet resuspended in 2 mL of FCS-DMSO freezing medium and stored at −80°C until analysis.

### Histone Modifications

Seven candidate genes were selected based on previous genetic association with helminth immunity: *IL4* and *IL13* at chr. 5q31 for their well-known involvement in helminth immunity (23, 24); *LIG4*/*ABHD13* and *TNFSF13B* in the *A. lumbricoides* susceptibility locus at chr. 13q33 (20) and *CHIA* at chr. 1p13.2, and *CHI3L1* at chr. 1q32.1 for their genetic association with anti-*Ascaris* IgE levels and protective immunity to helminths (21, 25). We also analyzed histone levels in the housekeeping gene *RPL32* encoding the Ribosomal protein L32 as a non-immune related control. Chromatin immunoprecipitation (ChIP) followed by quantitative polymerase chain reaction (qPCR) using specific primers (Table 2) were used to assess histone H3 acetylation of K9 and K14 (H3Ac) and H4 acetylation of K5, K8, K12, and K16 (H4Ac) levels at the promoters of the selected loci, as described previously (26). H3Ac and H4Ac levels were expressed as percentage of the input control and corrected for the isotype control.

**TABLE 2:** Primers used for quantitative assessment of H3 and H4 histone acetylation by PCR following chromatin immunoprecipitation (ChIP).

Target	Forward primer	Reverse primer
Chitinase 3 like 1 gene (*CHI3L1*) promoter	AATTGTGCCCAGTTTCCACC	GGGCTTCTGGAGATGTGACT
Acidic chitinase gene (*CHIA*) promoter	CGGACACTGGACTTAAGTTGT	GAAGCTTTGGCACCGTCT
Interleukin 13 gene (*IL13*) promoter	TGTGGGAGATGCCGTGGG	TCTGACTCCCAGAAGTCTGC
Interleukin 4 gene (*IL4*) promoter	TGGGTAAGGACCTTATGGACC	GGTGGCATCTTGGAAACTGTC
TNF superfamily member 13b gene (*TNFSF13B*) promoter	TAAGGGTGGGCTTCTCAGAC	GGTTTGCTGGCATTTACCCT
DNA ligase 4 gene (*LIG4*) promoter 1/Abhydrolase domain containing 13 gene (*ABHD13*) promoter^†^	GGCTCCCACATAACCTGTTC	GGTACGGAACTGGAGGGAGT
Ribosomal protein L32 gene (*RPL32*, *L23*; control gene)	GGAAGTGCTTGCCTTTTTCC	GGATTGCCACGGATTAACAC

*^†^See Kuhmann et al. (44) for the location of the promoters.*

### IgE Levels

Total serum IgE levels, specific IgE levels to the nematode *Ascaris*, and specific IgE levels to *Blomia tropicalis* and *D. pteronyssinus* were measured by ImmunoCAP (Thermo Fisher, Uppsala, Sweden). Total IgE levels were reported in IU/mL. Specific IgE levels above 0.35 kU_*A*_/L were considered positive for IgE sensitization. Specific IgE levels to the purified nematode specific marker ABA-1 were determined by indirect ELISA as described previously (27). ABA-1 is an allergen of *Ascaris* sp., and a member of the nematode polyprotein allergen/antigens with fatty acid-binding properties (28). It has been found only in nematodes (29) and has been used as a serological marker of Ascaris infection (14, 16).

### Statistical Analysis

Demographic data were compared between study subgroups by either Fisher’s exact test (binary variables) or Mann–Whitney *U* test (continuous variables). The correlation between acetylation levels with age, total egg burden, total IgE and specific IgE levels was calculated by Spearman correlation. Generalized linear models (GLM) were applied using the most appropriate function according to the distribution of the data to evaluate the relationship between histone acetylation and egg burden or IgE antibodies adjusting by the effect of age. Logistic regression was applied to model the relationship between histone acetylation and IgE sensitization to HDM as a categorical variable adjusting for age and gender. A *p*-value < 0.05 was considered significant. Model-based receiver operating characteristic (ROC) curves were drawn to test for the ability to predict sensitization to HDM allergens and the area under the curve (AUC) was calculated as a measure of performance using the logistic regression model described above.

## Results

### Study Population

The descriptive characteristics of infected and non-infected subjects are presented in Table 1. Of the twenty-one subjects infected by *A. lumbricoides*, nineteen were also infected by *Trichuris trichiura*. Median total IgE levels in infected subjects [902 IU/ml (IQR: 246-2097)] were higher than in non-infected [170 IU/ml (50.8–486), Mann–Whitney *p* = 0.003]. Median specific IgE to *Ascaris* spp. were significantly higher in the infected group [2.10 kU/l (0.57–8.72)] compared to non-infected [0.30 kU/l (0.08–1.14), Mann–Whitney *p* = 0.004], reflecting induction of the type 2 inflammation by the active helminth infection. Specific IgE levels to *Ascaris* significantly correlated with fecal egg counts of *A. lumbricoides* (rho 0.36, *p* = 0.02) and *T. trichiura* (rho 0.42, *p* = 0.007), thereby the sum of eggs of both helminths per individual was computed as *total egg burden* and this variable used in all subsequent analyses. This tropical population is also perennially exposed to HDM. A positive IgE response to *B. tropicalis* was detected in 43.9% of the individuals and to *D. pteronyssinus* in 36.6%. There was no difference in IgE levels to HDM between infected and non-infected subjects (Table 1).

### Histone Acetylation and Nematode Infection

Age was inversely correlated with total egg burden (rho − 0.40, *p* = 0.01) and directly correlated with H4Ac (rho 0.38, *p* = 0.014) and H3Ac (rho 0.30, *p* = 0.05) levels in *LIG4/ABDH13*. Total egg burden was inversely correlated with H4Ac levels in *IL13* (rho − 0.32, *p* = 0.03) and in *LIG4/ABDH13* (rho − 0.31, *p* = 0.04). To further evaluate these relationships considering the effect of age as confounding factor we implemented multivariate GLM. These analyses confirmed that increased H4Ac levels in *IL13* were associated with reduced total egg burden even after adjusting by age (Table 3). We also detected significant differences in the H3Ac acetylation levels and total egg burden in these genes, which remained significant after adjustment for *IL13* [β−0.58 (−0.86 to −0.29), *p* < 0.0001] and *LIG4/ABDH13* [β−0.28 (−0.40 to −0.16), *p* < 0.0001].

**TABLE 3:** Generalized linear regressions on the relationship between H4 acetylation with indicators of parasite and HDM exposure (*n* = 41).

	β (95% CI), *p*-value (crude)	β (95% CI), *p*-value (adjusted by age)
*Gene*	*Total egg burden*	

*IL13*	−0.21 (−0.32 to −0.09), *p* < 0.0001	−0.20 (−0.32 to −0.09), *p* < 0.0001
*LIG4/ABDH13*	−0.17 (−0.29 to −0.04), *p* = 0.009	−0.12 (−0.29 to −0.05), *p* = 0.17

	IgE to *Ascaris*	

*IL4*	0.29 (0.03−0.55), *p* = 0.03	0.32 (0.05−0.60), *p* = 0.02
*CHI3L1*	0.28 (0.05−0.51), *p* = 0.018	0.29 (0.08−0.51), *p* = 0.008

	IgE to *B. tropicalis*	

*TNFSF13B*	0.57 (0.32−0.82), *p* < 0.0001	1.50 (0.67−2.32), *p* < 0.0001
*LIG4/ABDH13*	0.69 (0.14−1.25), *p* = 0.014	5.36 (−6.9 to 17.6), *p* = 0.39

	IgE to *D. pteronyssinus*	

*TNFSF13B*	0.52 (0.28−0.76), *p* < 0.001	0.51 (0.26−0.76), *p* < 0.001
*LIG4/ABDH13*	0.53 (0.30−0.77), *p* < 0.001	0.47 (0.24−0.70), *p* < 0.001

### Histone Acetylation and IgE Levels to Ascaris

Specific IgE levels to *A. lumbricoides* extract were directly correlated with H4Ac (rho 0.35, *p* = 0.025) and H3Ac (rho 0.33, *p* = 0.03) levels in *IL4*. In addition, specific IgE levels to *A. lumbricoides* correlated with H4Ac in *CHI3L1* (rho 0.31, *p* = 0.049). The associations of the increased H4Ac levels in *IL4* and *CHI3L1* with increased IgE levels to *A. lumbricoides* also remained significant after adjusting by age (Table 3). Moreover, specific IgE levels to the nematode specific marker ABA-1 were directly correlated with histone acetylation levels in *CHI3L1*, affecting H4Ac (rho 0.38, *p* = 0.01) and H3Ac (rho 0.33, *p* = 0.03), supporting that epigenetic changes in these loci were induced by the infection with this nematode.

### H4 Acetylation and IgE Sensitization to HDM

**FIGURE 1:**
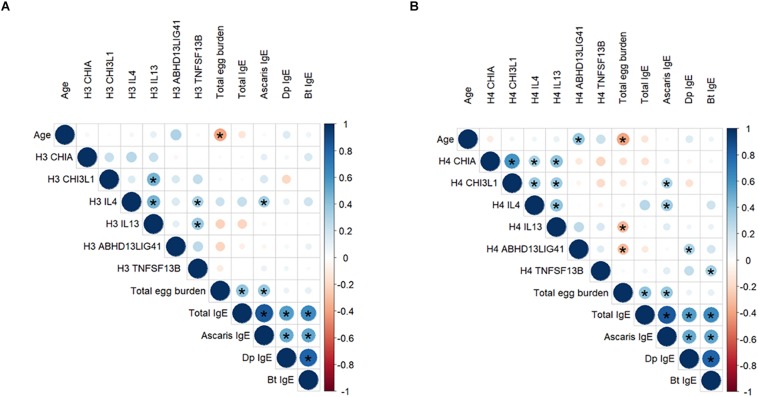
Correlation of H3Ac **(A)** and H4Ac **(B)** levels at six gene promoters with age, total egg burden, and total and specific IgE levels to Ascaris and HDM. The scale indicates the Spearman coefficient (Rho) from –1 to 1. Direct correlations are indicated in the blue scale and inverse correlations are indicated in the orange scale. Significant correlations (*p* < 0.05) are indicated with an asterisk. Dp: *D. pteronyssinus*; Bt: *B. tropicalis.*

**FIGURE 2:**
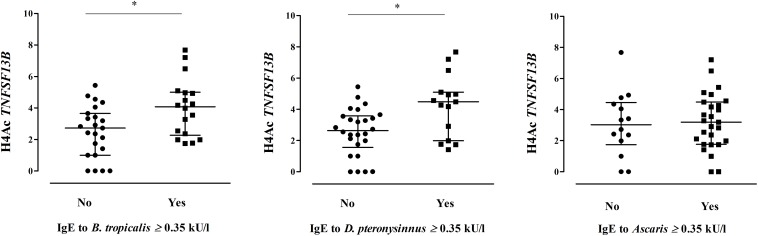
Comparison of the H4Ac levels in *TNFSF13B* according to HDM and *Ascaris* sensitization (*Mann–Whitney test *p* < 0.05). Each dot represents an individual, lines indicate median and IQR.

**FIGURE 3:**
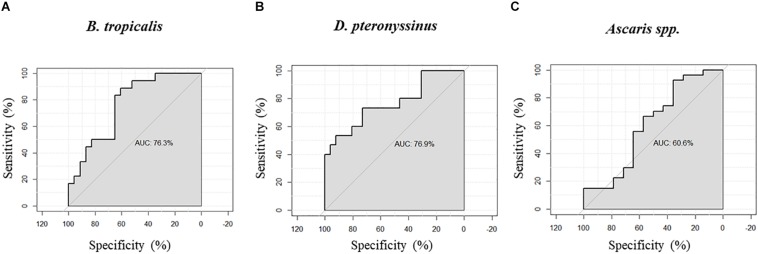
Receiver operating characteristic (ROC) curves and area under the curve (AUC) obtained from logistic regression models to predict the sensitization to HDM allergens. **(A)** H4 acetylation at the *TNFSF13B* gene versus IgE sensitization to *B. tropicalis*. **(B)** H4 acetylation at the *TNFSF13B* gene versus IgE sensitization to *D. pteronyssinus*. **(C)** H4 acetylation at the *TNFSF13B* gene versus IgE sensitization to *Ascaris* spp.

We found significant correlations between H4Ac levels in *LIG4/ABHD13* and *TNFSF13B* with specific IgE levels to HDM (Figure 1). GLM adjusting by age, confirmed the association between H4Ac levels in *TNFSF13B* with specific IgE levels to *B. tropicalis* and *D. pteronyssinus* (Table 3). When sensitization was analyzed as a categorical variable, H4Ac levels in *TNFSF13B* were significantly higher in individuals sensitized to HDM (Mann–Whitney test *p* < 0.05, Figure 2). This association remained significant after adjustment by age and gender in a logistic regression model. Next, we computed model-based ROC curves to see how well the regression models can predict sensitization to HDM allergens and computed AUC as a measure of performance. These analyses suggested a good predictive value of H4 acetylation over HDM sensitization with an AUC = 0.76 (Figure 3). Interestingly, the differences in the H4Ac in *TNFSF13B* gene were only detected with the response to HDM extracts but not with the *Ascaris* extract (Figures 2, 3).

## Discussion

The immune response to helminths and the allergic response share several biological pathways whose study have helped to understand the pathogenesis of both conditions. In the tropics, allergy and helminthiases are frequent, allowing the study of the mechanisms and clinical impact of their interactions (4). In addition to cellular and molecular mechanisms, genetic studies have provided evidence of genes involved in the same pathways activated in allergy and helminth immunity (19, 21). In contrast, epigenetic studies in this field are scarce, even considering the well-known importance of epigenetic mechanisms regulating gene expression. In this study, we present distinct epigenetic changes in *Ascaris* immunity and HDM IgE response. To our knowledge, this is the first report of association between differences in histone acetylation levels in the *IL13* gene and parasite egg burden, which is expected because the great importance of IL-13 in helminth immunity (30). Reduced egg burden can be explained because increased H4Ac in *IL13* may facilitate higher IL-13 production and suggests that this gene is sensitive to and modified by helminth infection. In our study asthmatic patients were not included; however, since IL-13 is also crucial for bronchial inflammation in asthma, this finding could help to explain why there is an increased severity of asthma in some Ascaris infected individuals (31, 32). Thus, our finding supports the traditional evolutionary hypothesis that Th2 allergic inflammation mechanisms are, at least partially explained, by helminth immunity legacy.

We also detected significant associations between H3 and H4 acetylation levels in the *LIG4/ABHD13* at the 13q33 locus with total egg burden, supporting previous association of this chromosomic region with Ascaris egg counts (20) and suggesting that susceptibility to this infection is not only mediated by genetic but also by epigenetic effects. The mechanisms how these genes participate in parasite immunity and influence egg burden remain to be elucidated. The role of IgE on protective immunity to Ascaris has not been sufficiently explored; however, considering previous associations between *LIG4/ABHD13* and the IgE responses to Ascaris (21), our results suggest that IgE may play a role in reducing egg burden during ascariasis (14, 16).

We also detected here a direct association between H4Ac in the *IL4* gene and IgE levels against *Ascaris* suggesting that increased acetylation at the *IL4* locus might influence IgE synthesis. H3 and H4 histone acetylation levels in *IL13* and *IL4* were directly correlated (Figure 1), however, the association between *IL13* acetylation with egg burden was significant with both histone marks (H3 and H4) while the association between *IL4* acetylation with specific IgE levels to *Ascaris* was only significant with H4Ac. The reason why each cytokine is associated to each of these outcomes is unknown, but a mice model revealed that a deletion in a DNase I-hypersensitive site 2 (HS2) element in the second intron of the interleukin 4 locus (*Il4*) impaired the acetylation of histone H3 at Lys9 and Lys14 in the *Il4* locus and affected the production of IL-4 but not of other Th2 cytokines, suggesting that it may occur chromosomal modifications on *Il4* that are independent of the *Il5* and *Il13* loci (33). Also in agreement with our previous genetic study (21), here we confirmed the association of *CHI3L1* with the specific IgE levels to *Ascaris* and ABA-1, showing for the first time increased H4Ac levels in high IgE responders to *Ascaris*. Recent studies revealed that expression of *CHI3L1* is modified upon contact with the *Ascaris* larvae (34). Further research is needed to elucidate the role of H4 acetylation in *CHI3L1* expression and its contribution to boosting IgE synthesis upon *A. lumbricoides* infection.

Epigenetic changes leading to bronchial inflammation and hyperresponsiveness have been induced by HDM under experimental conditions in mice (35, 36) suggesting that those epigenetic mechanisms may also contribute to asthma pathogenesis. Performing this kind of research in humans is more difficult, however indirect analyses can be done using specific phenotypes in natural exposed individuals (37). In this study we found that increased H4Ac at the gene *TNFSF13B* encoding B-cell activating factor was associated with elevated IgE levels to HDM allergens, suggesting that perennial exposure to HDM might affect histone acetylation at this locus in those predisposed to IgE sensitization. The B cell activating factor plays a critical role in B cell development and immunoglobulins production (38, 39). We found no association between H4Ac at this gene and IgE to Ascaris, which is in contrast with a previous study suggesting that this gene is associated with the humoral responses to Ascaris extract (40). However, in a more detailed study we found that among 13q33.3 region-genes enriched in high responders to Ascaris, *TNFSF13B* was not associated with specificity but rather the strength of IgE levels (21). These findings indicate that more studies are needed to dissect the control of the IgE response by the Ascaris susceptibility locus chr. 13q33.3.

In this study we evaluated acetylation changes in amino acid residues of H3 and H4; both implicated in the regulation of cytokine gene expression (41, 42). Whether histone tails act independently or have synergistic effects is still disputed. Acetylation of histone H4 is often found to be anticorrelated with acetylation of H3 or the other histones in binding of transcription factors, expressing genes or remodeling the chromatin (42, 43). In our study we found significant direct correlations of H3Ac and H4Ac levels in *IL-4, CHIA* and *CHI3L1* genes, while there were no significant correlations in the acetylation levels of these histones in *IL-13* and the two regions in 13q33 (Supplementary Figure S1). Interestingly, the associations with total egg burden were detected with both H3 and H4 histones while the associations with IgE levels only remained significant with H4Ac levels, suggesting that H4 marks might be more informative for epigenetic effects associated to the allergen exposure at these genes. We also showed for the very first time the significant correlations between the H4 acetylation levels in gene regions that albeit being in different chromosomes seem to be related (Figure 1B). Still, how acetylation levels at chitinase related genes in chromosome 1 are mechanistically connected to acetylation levels in *IL13* and *IL4* at the chromosome 5q31 remains to be investigated (Figure 1B), but the results suggest that nematode infection may induce coordinated histone changes in type 2 immunity pathways. However, since our study is cross-sectional, the effects of other environmental factors on the acetylation patterns cannot be ruled out.

In conclusion, this study provides evidence that allergen exposure alters the patterns of epigenetic modifications in human mononuclear leukocytes. Increased H4 acetylation in key immune genes is reflected by increased IgE levels to nematode and HDM allergens, suggesting an additional explanation to the similarities between helminth immunity and the allergic response. Further studies are needed to elucidate the functional effects of these acetylation marks on gene expression and the mechanisms promoting the type-2 immune response.

## Data Availability Statement

The datasets generated for this study are available on request to the corresponding author.

## Ethics Statement

This study was approved by the Ethics Committee of the University of Cartagena (nr. 1705-2012) and was conducted following the guidelines of the Declaration of Helsinki. All the participants gave their written informed consent prior to their inclusion in the study. Written informed consent to participate in this study was provided by the participants’ legal guardian/next of kin.

## Author Contributions

LC and DP conceived and designed the experiments. JZ, HH, and LE performed the experiments. JZ and NA analyzed the data. JZ, NA, HH, DP, HR, and LC contributed the reagents, materials, and analysis tools. NA, LC, and JZ wrote the manuscript. All authors involved in writing, reviewing, and editing. LC, JZ, HR, and DP involved in funding acquisition. LC overall responsibility of the project.

## Conflict of Interest

The authors declare that the research was conducted in the absence of any commercial or financial relationships that could be construed as a potential conflict of interest.

## Supplementary Material

FIGURE S1 Correlations between H3 and H4 acetylation levels in the six promoter regions analyzed in this study.
