# Supplementary figures and images for: IgE Levels to Ascaris and House Dust Mite Allergens Are Associated With Increased Histone Acetylation at Key Type-2 Immune Genes
Source: Front Immunol. 2020 Apr 28;11:756. doi: 10.3389/fimmu.2020.00756 (PMC7204827; doi:10.3389/fimmu.2020.00756)

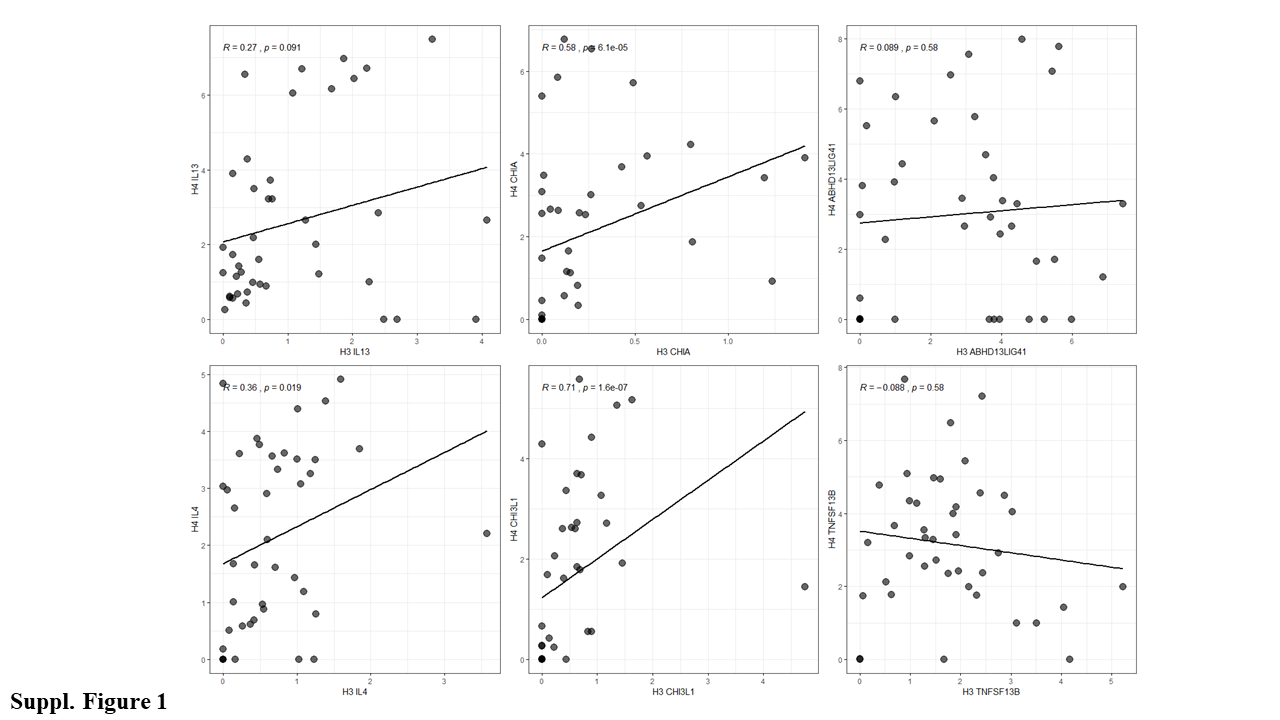

Supplement: FIGURE S1 — Correlations between H3 and H4 acetylation levels in the six promoter regions analyzed in this study. [file Image_1.TIF]
